# Notes

**Published:** 1897-01

**Authors:** 


### Notes.

The American Association of Obstetricians and Gynecologists at its ninth annual meeting, held at Richmond, Va., elected the following named officers for the ensuing year: President, James F. W. Ross, M. D., Toronto; Vice-Presidents, George Ben Johnston, M. D., Richmond, and John C. Sexton, M. D., Rushville, Ind.; Secretary, William Warren Potter, M. D., Buffalo; Treasurer, Xavier O. Werder, M. D., Pittsburg; Executive Council, Charles A. L. Reed, M. D., Cincinnati; Lewis S. McMurtry, M. D., Louisville; A. Vander Veer, M. D., Albany; J. Henry Carstens, M. D., Detroit; and William E. B. Davis, M. D., Birmingham. The next annual meeting was appointed to be held at the Cataract House, Niagara Falls, N. Y., Tuesday, Wednesday, Thursday, and Friday, August 17, 18, 19, and 20, 1897.

---

### CHANGE OF NAME.

The editors of *Mathews' Medical Quarterly* announce that with the January issue of that publication its name will be changed to *Mathews' Quarterly Journal of Rectal and Gastro-Intestinal Diseases.* This is a change which has been deemed necessary for some time, as it is essential that the title of a medical journal should convey to the reader an idea of its contents, and this has not been the case with its name from the beginning. There will be no change in the policy of the journal in the least. As it will continue to be the only English publication devoted to diseases and surgery of the rectum and gastro-intestinal tract, the articles which will appear in it will be limited to these subjects. The journal will continue to be edited by Drs. J. M. Mathews and Henry E. Tuley, and published in Louisville, Ky.

---

The Record would be much pleased to furnish notes of alumni of the Atlanta and Southern Medical Colleges. Send in your address and let your old college mates know how the world has served you.

THE Antikamnia Co. is remembering its friends by the presentation of a beautiful calendar, illustrated by Dr. L. Crusius. The pictures are of skulls with so varying expressions as to arouse wonder that there could be any semblance of life wrought upon the emblem itself of death.

---

PROF. ONICK, of the Technological School, has been making some very interesting skiagraphs in this city. He recently demonstrated before the class at the Southern Medical College by means of the X-ray a bullet next the chest wall. He has also successfully located fractures of different bones in several instances.

---

THE election of Dr. George Ben Johnston, of Richmond, Va., to the Presidency of the Virginia Medical Society and the Vice-Presidency of the American Association of Obstetricians and Gynecologists is a deserved compliment and a source of gratification to his many friends and admirers.

---

AT the last meeting of the Atlanta Society of Medicine the following officers were elected: George H. Noble, M. D., President; L. H. Jones, M. D., Vice-President; L. B. Grandy, M. D., Treasurer (re-elected); T. H. Hancock, M. D., Secretary.

---

OWING to the publication in the last issue of the proceedings of the Tri-State Medical Association, much matter was crowded out, which should have appeared in it.

---

DR. DUNBAR ROY was married December 16, at Richmond, Va., to Miss Carrie Ellis. We tender our best wishes and congratulations to the happy pair.

---

SINCE the death of Dr. Brooks, the editorship of the *Texas Courier-Record of Medicine* is in charge of Dr. J. O. McReynolds.

---

DR. FLOYD W. MCRAE has been elected vice-president of the Southern Surgical and Gynecological Association.

---

The New York Academy of Medicine will celebrate its fiftieth anniversary January 29.

